# Detecting signs of deterioration in young patients with serious mental illness: a systematic review

**DOI:** 10.1186/s13643-021-01798-z

**Published:** 2021-09-17

**Authors:** Lindsay H. Dewa, Arturas Kalniunas, Stephen Orleans-Foli, Sofia Pappa, Paul Aylin

**Affiliations:** 1 NIHR Patient Safety Translational Research Centre, Imperial College London, London, UK; 2 School of Public Health, Imperial College London, London, UK; 3 West London NHS Trust, London, UK; 4 Division of Psychiatry, Imperial College London, London, UK

**Keywords:** Serious mental illness, Mental health, Young adults, Deterioration, Indicators

## Abstract

**Background:**

Serious mental illnesses (SMI) such as schizophrenia and bipolar disorder first develop between ages 14 and 25. Once diagnosed, young peoples’ health can deteriorate, and it is therefore vital to detect this early to prevent severe outcomes including hospitalisations and deaths by suicide. The main study aim is to describe and discuss observational studies that examine signs of deterioration in young patients with SMI.

**Methods:**

A systematic review guided by the published protocol was conducted. Cumulative Index to Nursing and allied Health Literature (CINAHL), MEDLINE, Embase, PsycINFO, Health Management Information Consortium (HMIC) and Web of Science were searched against pre-defined criteria until 1 March 2021. Observational studies were extracted according to design, country, participant, indicator, outcome and main finding categories. Quality was assessed independently using the Newcastle Ottawa Scale (NOS).

**Results:**

Of the 15,788 publications identified, 5 studies were included and subjected to narrative synthesis. Two indicators of mental health deterioration were identified: cognitive functioning (decline, worsening and poor school/academic performance) and expressed emotion status. Indicators revealed mixed views on predicting deterioration. Worsening cognitive functioning and expressed emotion status significantly predicted medication non-adherence and relapse respectively. However, a decline in cognitive functioning (poor academic performance) was not found to significantly correlate to deaths by suicide. Study quality was mostly poor and associations between indicators and varied outcomes were weak. The heterogeneous nature of the data made comparisons difficult and did not allow for further statistical analysis.

**Conclusion:**

To our knowledge, this is the first review of observational studies to identify indicators of deterioration in young patients with SMI. Worsening cognitive functioning and expressed emotion status could indicate non-adherence and relapse in young patients with SMI but larger sample sizes in good quality studies are needed. The dearth of observational studies means further research is required to ascertain other indicators of deterioration before serious outcomes occur.

**Funding:**

This work was supported by the National Institute for Health Research (NIHR) Imperial Patient Safety Translational Research Centre via an NIHR programme grant. The authors are also grateful for support from the NIHR under the Applied Health Research programme for North West London and the NIHR Imperial Biomedical Research Centre. The views expressed in this publication are those of the authors and not necessarily those of the National Health Service (NHS), the NIHR or the Department of Health. The funders had no role in study design, data collection and analysis, decision to publish, or preparation of the manuscript.

**Trial registration:**

This systematic review has been registered on PROSPERO (registration number: CRD42017075755).

**Supplementary Information:**

The online version contains supplementary material available at 10.1186/s13643-021-01798-z.

## Background

Serious mental illnesses (SMI) can be debilitating, affecting behaviour, day-to-day functioning and mood. Three quarters of SMIs, including schizophrenia and bipolar disorder first develop in young people, from 14 to 25 years [1]. The exact prevalence for this age group is unknown, but an estimated 0.5% of young people suffer from a psychotic disorder and 3.4% from bipolar disorder [2]. This is a critical stage for young people because of the neurological, biological and cognitive changes of adolescence to young adulthood, in addition to environmental changes (e.g. major educational milestones in secondary and tertiary education, employment, increased social demands and new relationships) [3, 4]. For those receiving care for their condition, it is also likely a difficult transition from children and adolescent (CAMHS) to adults’ mental health services (AMHS) [4, 5]. Early interventions or preventative strategies in this period are crucial as they offer a potential opportunity to mitigate the stressors that impact on physical, emotional and psychological wellbeing [6, 7]. However, the non-recognition of early signs of deterioration, absence of, or delay in accessing appropriate mental health services coupled with the reluctance for young people to disclose impromptu concerns to healthcare professionals [8], means young people are still at potential risk of deteriorating in mental state [9, 10]. Outcomes can include admission to emergency care and participating in unsafe behaviours (e.g. aggression, self-harm, death by suicide).

Intervening at the earliest sign of deterioration in a young person is therefore paramount. Deterioration can happen in both mental and physical health that becomes progressively worse over time [11]. Risk factors for deterioration in people with SMI resulting in inpatient admission are well known, and include sedentary behaviour, psychosis, aggression, suicidal thoughts and behaviour and mania [12–16]. Whilst some of these signs can be difficult to detect and manage, less severe signs of deterioration could potentially give an earlier indication of future deterioration. Examples include insomnia and hypersomnia, mood changes and reduced activity (e.g. staying in bed, not leaving the house) [17, 18]; in young people, sometimes misinterpreted as “normal adolescence”. Similarly, signs of physical health deterioration in people with SMI include pain, substance misuse and respiratory issues [19]. Monitoring and detecting less severe signs that include these everyday changes in mental state, behaviour or cognition, may allow for earlier intervention and prevention of the potential detrimental sequelae of a full relapse by offering alternative strategies that are likely to improve long-term prognosis.

Only one report, recently updated, has reviewed deterioration in mental state, which was not restricted by age [20, 21]. The updated report sought to identify tools to detect deterioration since the previous report [21]. In addition, the authors also reported five key indicators of mental state deterioration which derived from a collaborative consensus building exercise with clinicians and people with lived experience. These indicators included reported change for the worse in mental state, distress, loss of touch with reality or consequences of behaviours, loss of function and elevated risk to self, others or property [21]. Whilst the report was extensive, it did not focus on or highlight findings in young patients or people with serious mental illness, nor did it cover physical deterioration. This review was also scoping in nature therefore further underscoring the need to conduct a more systematic review of the existing literature on detecting indicators for physical and mental health deterioration in relation to young patients with SMI.

The study aim was to describe and discuss observational studies that examine deterioration in young patients with SMI. Specifically, we wanted to (i) identify indicators of deterioration over time, (ii) critically reflect on the quality of studies and (iii) assess the evidence associated with these indicators to detect deterioration. It will also highlight the gaps in knowledge to inform future research.

## Methods

This review was guided by Centre for Reviews and Dissemination’s guidelines [22] and reported in accordance with PRISMA guidelines [23] (Supplementary file 1). Our review protocol, including our search strategy was peer-reviewed, published [11] and registered with PROSPERO in September 2017 (reg: CRD42017075755).

### Search strategy and study selection

We searched Cumulative Index to Nursing and Allied Health Literature (CINAHL), MEDLINE, Embase, PsycINFO, Health Management Information Consortium (HMIC) and Web of Science databases against a list of pre-defined comprehensive search terms on 7 November 2017 and updated on 1 March 2021 to search for more recent papers. Searches were run independently in each database to reflect their individual set-up, MESH terms and relevant subject headings (Supplementary file 2). The first 10 pages of Google were also searched for grey literature.

The search strategy was developed iteratively and approved by two members of the initial team (LD, PA), two institutional librarians and external peer-review. The search terms were based on five main facets: age, serious mental illness, sign, deterioration and patient. Whilst all effort was made to keep to the original search strategy in our protocol [11] some changes were necessary. For example, we found that our original age bracket of 18–25 years old was too restrictive, as no papers used this bracket. Instead, the age bracket was widened to 15–30, which was approved by the initial research team (LD, PA, SOF).

Papers were selected if they met the followed inclusion criteria (a) observational studies with definitive time points (b) population of young patients (15–30 years old) with a SMI including schizophrenia, major depression disorder, bipolar disorder and other related psychotic disorders and (c) focused on detecting mental or physical deterioration. Comprehensive definitions of all facets are including in the protocol [11]; however, we felt it was useful to reiterate briefly here. Deterioration was defined as change in mental state or clinical state over time that led to an adverse outcome. Subsequent indicators of this deterioration could include mood, behaviour, affect, thought, perception or cognition.

Exclusion criteria were (a) reviews, conference abstracts, book chapters, protocols, editorials, opinion, discussion pieces, commentaries and case reviews, (b) young patients without a SMI and (c) non-English language.

All title and abstracts were screened by one reviewer (LD) in COVIDENCE. Ten percent were independently screened by another (AK). Two reviewers (AK, LD) then independently screened all full-text papers. Inter-rater agreements were calculated using the Kappa statistic and performed in Statistical Package for Social Sciences (SPSS Statistics 25). Any disagreements were resolved in separate meetings with two reviewers (AK, LD). Finally, reference lists of all included papers were also screened and papers that matched inclusion criteria were added.

### Data extraction

Three studies were subjected to an initial extraction pilot as recommended. Author, year of publication, country, observational design, setting, sign of deterioration, outcome and main results. Studies were independently extracted by two reviewers (AK, LD) and then compared to see if extraction was similar. Observation revealed data was inline and therefore we proceeded to extract the remaining information independently. Discussion on disagreements with third party (PA) was not needed.

### Quality assessment

The data was critically appraised by two independent reviewers (AK, LD) using the Newcastle-Ottawa Scale (NOS) [24]. It is recommended to assess quality for non-randomised studies (i.e. cohort and case-control studies) [25]. The scale had nine possible ‘stars’, which could be assigned across three sections: (i) selection of study groups (4 stars), (ii) comparability of the groups (2 stars) and (iii) whether the anticipated outcome had been obtained (3 stars). Studies were assigned as poor, fair or good quality (Table 1). Good studies required 3 or 4 in selection AND 1 or 2 in comparability AND 2 or 3 in outcome. Fair studies required 2 in selection AND 1 or 2 in comparability AND 2 or 3 in outcome. Finally, poor studies needed 0 or 1 in selection OR 0 in comparability OR 0 or 1 in outcome. Studies were not excluded based on quality in order to give a thorough overview of the area.

**Table 1:** Summary of included papers

Author and country	Design	Participants	Indicator of deterioration	Outcome measure	Main findings	NOS assessment score
Cotton et al. [26] Australia	Cohort	661 Aged 15–29 years Schizophrenia	Cognitive functioning (decline/stable)	Treatment reduction or discontinuation Number of admissions	Decline in cognitive functioning predicts a higher number of hospital admissions	Poor
Gonzalez-Blanch et al. [27] Spain	Cohort	63 Aged 15–25 First episode psychosis	Expressed emotion status (high/low)	Cannabis use	Cannabis use was significantly related to high expressed emotion	Poor
Gunnell et al. [28] Sweden	Cohort	186808 Aged 16 Serious mental illness diagnosis varied	Cognitive functioning (poor school performance)	Deaths by suicide	Poor school performance at aged 16 was not significantly associated with suicide risk	Good
Lambert et al. [29] Australia	Cohort	786 Aged 15–29 First-episode psychosis	Worsening cognitive functioning	Medication-adherence/refusal	Non-adherent patients had worse outcomes. More likely to be disengaged from services, not in remission of symptoms and have worse severity of illness.	Poor
Linszen et al. [30] Netherlands	Cohort	97 Aged 15–26 years Schizophrenia, schizoaffective disorders, schizophreniform disorders and other psychotic disorders	Expressed emotion status (high/low)	Relapse	Five times higher risk of relapse in those who experienced high expressed emotion	Poor

*NOS* Newcastle Ottawa Scale

### Data synthesis

As expected, the included studies were heterogeneous and a meta-analysis was not possible [11]. There is no framework for narrative synthesis for observational studies; however, we chose to further synthesise data using the general framework for systematic reviews [31]. The framework includes (i) developing a theory of how an intervention works, why and for whom; (ii) developing a preliminary synthesis of findings of included studies (through groupings, clustering and vote counting); (iii) exploring relationships within and between studies (through an evidence map); and (iv) assessing the robustness of the synthesis (e.g. reflecting critically on the synthesis process). As our review focused on observational studies, the ‘developing a theory’ element was not appropriate and was removed, in line with guidance [31] and other studies [32].

### Patient and public involvement (PPI)

Involving patients or the public in systematic reviews is recommended and beneficial to conducting a systematic review [33]. We selected three people with experience of mental illness and participants in a previous study [34] to inform our review in a 2-h face-to-face meeting. More detail on our PPI can be found our protocol [11] but in brief, they helped guide (i) the search terms, (ii) research question and (iii) selection criteria. Their involvement resulted in a focus on young patients and the research question changing from “What are the indicators of deterioration in mental healthcare?” to “What are the signs of deterioration in young adults with serious mental health problems?”.Their thorough understanding of deterioration helped inform the researchers and their account of deterioration (e.g. sleeping too much, isolation) was incorporated into the paper introduction. Moreover, they were happy with the use of the word deterioration.

## Results

**Fig. 1:**
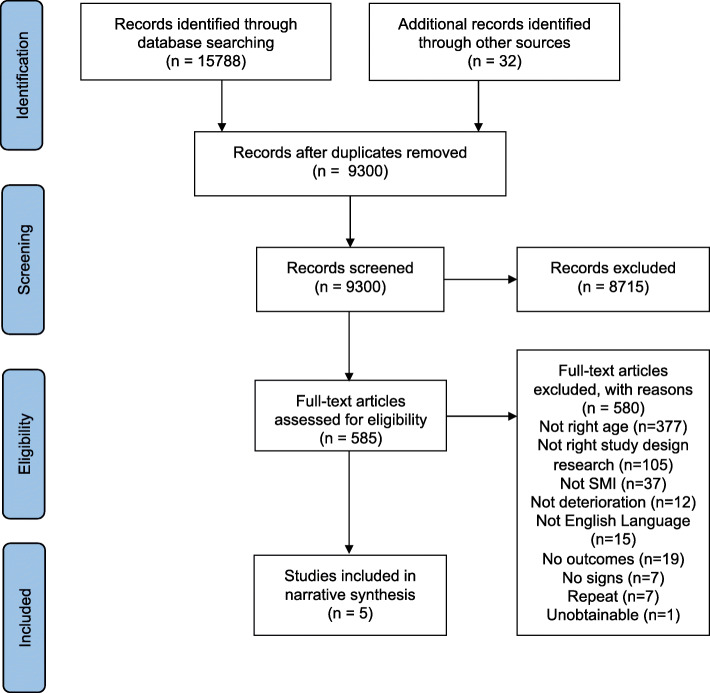
PRISMA diagram

We identified 15,788 publications from the searching stage and subsequently screened 9300 title and abstracts after duplicates were removed (Fig. 1). Ten percent screened by an independent reviewer showed strong agreement (*K* = .91). All 585 full-text publications were screened by two independent reviewers; our agreement was also substantial (99.7% agreement and *K* = .75). Subsequently, only five publications matched our selection criteria and were included. The NOS quality assessment score ranged from four to nine, with four studies scoring as poor and one as good (Table 1). Whilst some studies controlled for some important variables three did not control for usual factors such as age, gender and socio-demographics.

### Developing a preliminary synthesis of findings of included studies

Three studies were conducted in Europe [27, 28, 30] and two in Australia [26, 29]; all used a cohort design. However, the method of data collection varied; three used prospective data linkage [26, 28, 29] and two used a mixture of self-report standardised measures and clinical structured interview [27, 30]. Of the 5 studies, 188,234 patients participated (96,014 males and 91,559 females; 661 gender comparison missing). Mean follow-up duration was 6.4 years (ranged from 7 months to 17 years).

Gender differences were reported in two studies [28, 30] and ethnicity and sociodemographic status was only mentioned in one study [30]. SMI diagnoses varied and included schizophrenia, schizophrenia and schizoaffective disorder, schizoaffective disorder, affective psychoses, non-affective psychoses, unipolar depression, schizophreniform, major depressive episode with psychotic features, bipolar disorder and other psychotic disorders. All participants had had previous treatment. Outcomes also differed and included death by suicide [28]; cannabis use [27]; number of admissions [26]; treatment reduction or discontinuation [26]; medication refusal; medication non-adherence [29] and relapse [30]. Two indicators of deterioration were examined across five studies: cognitive functioning (decline, worsening or poor school performance) [26, 28, 29] and expressed emotion status (high/low and high/low criticism) over two-time points (Table 1) [27, 30].

In one study, young patients who had a decline in cognitive functioning were more likely to have an increased number of hospital admissions than those with stable functioning (OR 1.44 CI 1.16 to 1.78) [26]. Similarly, in another study, young patients who had early worsening cognitive functioning were more likely to either refuse (OR 0.94 CI 0.92 to 0.96) or poorly adhere to medication (OR 0.97 CI 0.95 to 1.00) [29]. However, only a weak association was demonstrated. Similarly, in another study, associations between poor school performance at aged 16 and suicide in females and males who were admitted to a psychiatric hospital over the follow-up period was also weak (HZ 1.3 CI 0.9 to 1.8 and HZ 0.8 CI 0.6 to 1.0 respectively) [28].

One study examined expressed emotion but included different outcomes. For example, González-Blanch and colleagues identified expressed emotion status (high criticism) as a sign of deterioration that predicted cannabis use [27]. In contrast, Linszen and colleagues examined expressed emotion status (high) as a predictor of relapse. Expressed emotion status (high/high criticism) had a stronger association in predicting relapse than cannabis use [27, 30].

### Exploring relationships within and between studies

Most studies centred on examining ‘within study’ relationships as part of their study aim. Therefore, most relationships are reported as main findings above. One additional within-groups finding in one study followed-up cannabis scores over time as a continuous variable across the expressed emotion groups (low/high criticism) [30]. Cannabis use increased in the high criticism group at 7 months whereas it decreased in the low criticism group [27]: however, these changes were not significant. Additional ‘between studies analysis’ across included studies was not possible due to differing statistical analysis.

### Assessing the robustness of the synthesis

**Fig. 2:**
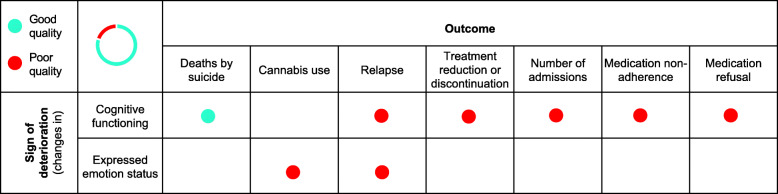
Evidence map

Best evidence synthesis was not possible as there were only five studies, and most were of poor quality (Fig. 2). Therefore, we did not remove studies based on study quality in order to give a better understanding of what has been done and identify research gaps. Based on the analysis of relationships between and within studies described above the strength of evidence regarding early signs of deterioration in young people with SMI is weak. The reasons for this are multifaceted. First, studies were mostly of poor quality. In general, they failed to adequately control for confounding factors and either ascertainment of exposure or outcome was done via self-report. Second, the included papers did not fully centre on decline or deterioration in mental state as their primary outcome making it difficult to extract information directly relating to our research question. Therefore, the papers did not always provide the necessary detail to allow for sufficient data to be utilised. Third, whilst level of agreement was high at the abstract/title stage (*K* = .91) and agreement was moderate at the full-text discussion phase of included studies (*K* = .75). Finally, authors were not blind to names, institutions and journal upon bias assessment.

## Discussion

### Key findings

To our knowledge, this is the first systematic review on the detection of early signs of deterioration in young patients with SMI. We only found five papers, with the main focus on young patients with schizophrenia (or with psychosis). Our systematic approach, guided by our protocol, meant that we would have excluded birth cohort which would have likely increased the number of included studies. However, following people from birth was outside the focus of this review. Nevertheless, the paucity of papers in general resulted in incorporating studies mostly reporting on patients with prodromal signs of mental illness rather than on deterioration in the context of SMI. However, this highlights a critical research gap. It may also suggest that patients with SMI are different to patients with common mental health disorders (e.g. mild to moderate anxiety and depression) and premorbid conditions in the context of detecting deterioration. It would be interesting to determine whether interventions for these groups improve outcomes for young people with SMI. Regardless, these patient groups are completely separate groups that deserve attention.

Most studies were of poor quality, included small sample sizes and analysed deterioration using a between-group analysis across two separate groups (e.g. decline in/stable cognitive functioning, non-adherence/adherence, low/high expressed emotion and low/high criticism) rather than showing change over time within-groups. The statistical analysis performed varied between studies making comparisons difficult. Two studies used hazard ratios (HZ), two used odds ratio (OR) and one reported to have used OR but failed to report OR statistics correctly. For example, Gonzalez-Blanch and colleagues did not give odds ratios or relative risks that would have shown some indication of risk and give us a better understanding of the size of the effect [27]. In another study, a number of errors were noted, for example, associated factors and outcomes were the wrong way round in the table (no acknowledgement) which limited the validity of the results. Moreover, we could only report on some data within the main context of the paper to answer our research question and some papers included a wide spectrum of mental illness that encompassed SMI without clear differentiation in the results; it was inappropriate to include other findings within our analysis. Overall, deterioration was difficult to determine in the included papers.

As outlined above, the previous report in this area identified five indicators of mental state deterioration [21]. In line with this report, cognitive functioning, was also found to be an indicator of deterioration in our review. In contrast, expressed emotion was not identified as a separate indicator in the previous report on its own. However, it could align with the “reported change” indicator [21] which would cover change in emotion generally. Other previous studies have focused on (i) risk factors for deterioration in young adults with mental illness or (ii) risk factors for relapse in adults with SMI that leads to inpatient admission [12–16]. Findings in our review demonstrate a lack of studies that examine subtle and less severe signs of deterioration in young patients with SMI. However, declining cognitive function was identified as a sign, and associated with poor outcomes in young patients with schizophrenia, adding to the strong evidence about this association [35]. Similarly, expressed emotion was identified as a predictor of relapse, in line with other studies [36].

### Strengths and limitations

To our knowledge, this is the first systematic review to examine indicators of deterioration in young patients with existing SMI. We performed a robust systematic search in line with PRISMA guidelines. However, there are clear limitations. We only included English language publications and our full-text screening inter-rater agreement was moderate despite high level of agreement in the previous stages. We expected to keep to the published protocol at all times however, the lack of papers in accordance with our original criteria meant adjustments were needed (e.g. extended age range). Moreover, the small number of papers and heterogeneous data meant meta-analysis was not possible.

### Implications for future research

This review highlighted the importance of recognising cognitive decline and expressed emotion as signs of deterioration in young patients with SMI. However, all eligible studies tended to use cohort designs with self-report or clinical judgement without prominent temporal statistical analysis. Future studies should examine cognitive decline, expressed emotion and other signs of mental, and physical deterioration including sleep problems, low mood and lack of activity, over long periods of time in large samples of young patients with an established SMI diagnosis, and also in those who less severe or common mental disorders as well as those who have not yet been formally diagnosed with a condition. Detecting signs as early as possible, in those without a diagnosis, can lead to more timely and appropriate interventions, reducing the need for lengthy psychiatric hospital stays, and improving serious outcomes (self-harm and suicide). However, this may be difficult in a young cohort who do not engage with healthcare professionals [8]. Research into the effectiveness of passive monitoring using technology (e.g. sleep-wake cycle, physical activity, phone usage) to detect deteriorating mental health, could be a potential targeted area of research as it does not require young people to engage with or access mental health services.

## Conclusions

There is a dearth of observational studies that examine indicators of deterioration in young patients with SMI. Expressed emotion status could indicate relapse in young patients with SMI but larger sample sizes are needed. Furthermore, high-quality longitudinal observational studies with temporal analysis are warranted to ascertain other indicators of mental and physical deterioration in young patients with SMI before serious outcomes occur.

## Supplementary Information

**Additional file 1:.** Supplementary file 1. PRISMA 2009 checklist.

**Additional file 2:.** Supplementary file 2. Example of search strategy used in MEDLINE.

## Data Availability

Not applicable

## Abbreviations

CAMHS: Children and Adolescent Mental Health Services
CINAHL: Cumulative Index to Nursing and Allied Health Literature
HZ: Hazard ratios
HMIC: Health Management Information Consortium
NIHR: National Institute for Health Research
NOS: Newcastle Ottawa Scale
OR: Odds ratio
PPI: Patient and public involvement
PRISMA: Preferred Reporting Items for Systematic Reviews and Meta-Analyses
SMI: Serious mental illness(s)
SPSS: Statistical Package for Social Sciences
