# Supplementary material for: Detecting signs of deterioration in young patients with serious mental illness: a systematic review
Source: Syst Rev. 2021 Sep 17;10:250. doi: 10.1186/s13643-021-01798-z (PMC8447694; doi:10.1186/s13643-021-01798-z)
Supplement: Supplementary file 2 — Additional file 2:. Supplementary file 2. Example of search strategy used in MEDLINE. [file 13643_2021_1798_MOESM2_ESM.docx]

**Supplementary method**

**Example of search strategy used in Medline**

Young Adult/ OR Adolescent/ OR young adult*.ti,ab,kw. OR adolescen*.ti,ab,kw. OR young person*.ti,ab,kw. OR young people.ti,ab,kw. OR youth*.ti,ab,kw. OR Transition to adult care/ OR ((transition* or transfer* or hand over or handover or handoff) adj5 (adult care or adult services)).ti,ab,kw.

**AND**

OR Bipolar Disorder/ OR bipolar disorder*.ti,ab,kw. OR exp Schizophrenia/ OR schizophrenia.ti,ab,kw. OR Psychotic Disorder/ OR psychotic disorder.ti,ab,kw. OR depressive disorder/ OR Depressive Disorder, Major/ OR major depression.ti,ab,kw. OR major depressive disorder.ti,ab,kw. OR depression/ OR depression.ti,ab,kw. OR schizoaffective.ti,ab,kw. OR psychosis.ti,ab,kw. OR mania.ti,ab,kw. OR serious mental illness*.ti,ab,kw OR severe mental illness*.ti,ab,kw.

**AND**

OR warn*.ti,ab,kw. OR MEWS.ti,ab,kw. OR predict*.ti,ab,kw. OR detect*.ti,ab,kw. OR sign*.ti,ab,kw. OR measure*.ti,ab,kw. OR gauge.ti,ab,kw. OR index.ti,ab,kw. OR criteria.ti,ab,kw. OR highlight*.ti,ab,kw. OR monitor*.ti,ab,kw. OR symptom*.ti,ab,kw. OR signal*.ti,ab,kw. OR diagnos*.ti,ab,kw. OR characteristic*.ti,ab,kw. OR alarm*.ti,ab,kw. OR alert*.ti,ab,kw. OR caution*.ti,ab,kw. OR forewarn*.ti,ab,kw. OR trigger*.ti,ab,kw. OR risk.ti,ab,kw. OR factor*.ti,ab,kw. OR indicat*.ti,ab,kw.

**AND**

OR declin*.ti,ab,kw. OR deteriorat*.ti,ab,kw. OR worse*.ti,ab,kw. OR downfall.ti,ab,kw. OR weak*.ti,ab,kw. OR descen*.ti,ab,kw. OR laps*.ti,ab,kw. OR dip*.ti,ab,kw.

**AND**

OR patient*.ti,ab,kw. OR inpatient*.ti,ab,kw. OR in-patient*.ti,ab,kw. OR outpatient*.ti,ab,kw. OR out-patient*.ti,ab,kw. OR exp Patients/ OR exp Inpatients/ OR Outpatients/
